# High-Efficiency Adsorption of PS, PE, and PP Microplastics from Environmental Waters Using a Cross-Linked Chitosan/Graphitic Carbon Nitride/ZIF-67 Nanocomposite

**DOI:** 10.3390/polym18151904

**Published:** 2026-08-03

**Authors:** Amr A. Yakout, Faten M. Ali Zainy

**Affiliations:** Department of Chemistry, College of Science, University of Jeddah, Jeddah 23890, Saudi Arabia

**Keywords:** microplastics, chitosan, graphitic nitride, ZIF-67, adsorption

## Abstract

Municipal wastewater is a major pathway for the continuous release of microplastics into aquatic environments, making the development of efficient and reusable capture materials essential for advanced water treatment. In this study, a multifunctional ZIF-67/*g*-C_3_N_4_/CS nanocomposite was designed by integrating cobalt-based zeolitic imidazolate framework ZIF-67 with graphitic carbon nitride (*g*-C_3_N_4_) and a chitosan (CS) biopolymer matrix. The novelty of this material lies in combining the high porosity and tunable surface chemistry of ZIF-67, the π-rich layered structure of *g*-C_3_N_4_, and the hydrophilic, amino-rich chitosan framework into a single adsorptive platform for simultaneous removal of chemically different microplastics. The nanocomposite achieved high removal efficiencies for polystyrene (PS), polypropylene (PP), and polyethylene (PE) microplastics with particle sizes of 20–25 μm, reaching 97.4%, 92.1%, and 90.3%, respectively, at pH 7.6 within 25 min. The higher affinity toward PS is attributed to additional π–π interactions between the aromatic PS chains and the conjugated domains of *g*-C_3_N_4_/ZIF-67, whereas PP and PE removal is mainly governed by hydrophobic adhesion, surface trapping, and interfacial interactions with the chitosan-supported porous framework. The equilibrium data were well described by both Langmuir and Freundlich models, with maximum adsorption capacities of 97.69, 94.86, and 93.67 mg g^−1^ for PS, PP, and PE, respectively. The nanocomposite retained high recyclability, maintaining 95–97 ± 3.1% removal after five adsorption–desorption cycles. These findings demonstrate that ZIF-67/*g*-C_3_N_4_/CS is a durable and high-performance adsorbent for microplastic remediation, with strong potential for application in municipal wastewater treatment, constructed wetlands, and advanced water-polishing systems.

## 1. Introduction

Microplastics (MPs) have emerged as pervasive contaminants in aquatic systems and are now recognized as a significant threat to both ecosystem integrity and human health [1,2]. They are continuously introduced into water bodies through multiple pathways, including stormwater runoff, river transport, industrial effluents, sewage discharge, wind transport, tidal exchange, and flood-driven redistribution [3,4]. Conventionally, MPs are defined as plastic particles smaller than 5 mm, and they are now widely detected in rivers, lakes, estuaries, and marine environments worldwide [5,6,7]. Their occurrence is particularly pronounced in densely populated urban regions, where intense anthropogenic activity accelerates the generation and release of plastic debris into water networks [8]. Because of their persistence, buoyancy, and resistance to natural degradation, microplastics can accumulate in aquatic habitats and impose chronic stress on aquatic organisms, thereby disrupting food webs and ecosystem functions [9,10]. Of equal concern is their entry into the human body, where they have been detected in blood, placenta, heart, and brain tissues, raising serious concerns regarding their possible association with neurological, cardiovascular, and systemic disorders [11,12]. In addition, microplastics may interfere with microbial communities and influence horizontal gene transfer in environmental systems [13].

Microplastics are generally classified as either primary or secondary particles according to their origin. Primary microplastics are intentionally manufactured at microscopic dimensions and are used in products such as cosmetics and personal care formulations, whereas secondary microplastics arise from fragmentation of larger plastic items under the action of sunlight, mechanical abrasion, oxidation, and weathering. Domestic wastewater is one of the principal routes through which both forms enter aquatic environments [14]. Among household sources, laundering of synthetic textiles is especially important, as fibers released from polyester, nylon, acrylic, and polyamide fabrics are transported directly into sewage systems. Additional contributions arise from personal care products and a wide range of domestic and industrial activities. For this reason, urban wastewater is considered a major conduit for the transfer of MPs from human activities to natural waters, and preventing this migration remains an urgent environmental priority [15,16].

A wide range of technologies have been explored for microplastic remediation, including chemical, biological, and physical treatment approaches. Chemical methods mainly involve advanced oxidation and photocatalytic degradation [17], whereas physical methods include adsorption, coagulation, and membrane filtration. Although coagulation can efficiently separate suspended plastic particles, the subsequent handling and disposal of the generated flocs remain problematic because improperly managed sludge may release the captured microplastics back into the environment [18]. Membrane-based processes generally offer high separation efficiency, but their practical implementation is frequently constrained by membrane fouling, material degradation, and high operational and maintenance costs [19]. Biological degradation is attractive from a sustainability perspective, yet its application to MPs remains limited by low efficiency and very slow degradation rates [20]. In contrast, adsorption is increasingly regarded as one of the most practical physical strategies for microplastic control because of its operational simplicity, low energy demand, flexibility, and potential for high removal efficiency.

A variety of adsorbents have therefore been investigated for MP capture, including activated carbon, biochar, aerogels, sponges, carbon nanotubes, and metal–organic frameworks (MOFs) [21,22,23,24,25]. Carbonaceous materials are particularly interesting because they can interact with MPs through surface functional groups as well as through adhesion, trapping, and entanglement within porous structures, often resulting in efficient removal [26,27]. However, purely carbon-based sorbents may suffer from limited selectivity, aggregation, and difficulties in separation and regeneration after use. These limitations have encouraged the design of hybrid materials in which carbonaceous components are integrated with functional polymers or porous coordination frameworks to improve adsorption performance and handling properties.

Among bio-based materials, chitosan (CS) has received considerable attention as an adsorptive platform for MP recovery [28,29]. Its attractiveness lies in its abundance, biodegradability, biocompatibility, and rich surface chemistry, particularly the presence of amino and hydroxyl groups that can be further modified to tune selectivity and interfacial affinity. In aqueous systems, chitosan can also act as a film-forming and structurally cohesive matrix, which makes it useful for constructing stable composite adsorbents. These features have stimulated growing interest in CS-based materials for the capture of MPs and other classes of water contaminants [28,29].

MOFs represent another important class of materials for pollutant capture because of their high surface area, tunable pore structure, and chemically tailorable surfaces. Their modular architecture, built from metal centers and organic linkers, allows systematic adjustment of pore environment, hydrophobicity, and interfacial functionality, which is highly advantageous for particulate pollutant capture. For microplastic and nanoplastic remediation, MOFs can provide abundant accessible adsorption domains and promote interfacial adhesion to polymer particles. However, the practical use of MOFs in water treatment is often limited by insufficient mechanical robustness, particle aggregation, and difficulties associated with recovery and reuse in aqueous media [30,31]. One effective way to address these limitations is to hybridize MOFs with biopolymers or other complementary phases, thereby combining the porosity and surface tunability of the MOF with the processability, flexibility, and water compatibility of the supporting matrix [30]. Such hybridization can create multifunctional interfaces capable of promoting particle capture through a combination of physical trapping, surface adhesion, hydrogen-bond-assisted interactions, electrostatic effects, and hydrophobic affinity.

Although MOF-based sponges and aerogel-like materials have achieved promising MP-removal efficiencies, their broader application remains open to question [32,33,34]. Foam- or sponge-type supports are often derived from polymeric materials that may degrade or fragment during use, thereby introducing the risk of secondary contamination. In addition, many such systems remain largely confined to proof-of-concept laboratory demonstrations. These concerns highlight the need for robust, reusable, and environmentally safer adsorbents that can operate efficiently in realistic wastewater matrices without introducing additional pollutant burdens.

In this context, we designed a ternary hybrid adsorbent based on ZIF-67/*g*-C_3_N_4_/CS for the efficient removal of chemically distinct microplastics from water. In this architecture, ZIF-67 was selected as the cobalt-based MOF because of its porous imidazolate framework, high accessible surface area, and ability to provide favorable interfacial affinity toward polymer particles. *g*-C_3_N_4_ was incorporated as a layered, π-conjugated component capable of enhancing surface roughness, interfacial contact, and affinity toward aromatic polymer structures, particularly polystyrene. Chitosan was used as the biopolymeric matrix to provide hydrophilicity, amino- and hydroxyl-rich surface chemistry, and structural cohesion, while also improving dispersibility and operational stability of the hybrid material in water. The novelty of the present work lies in the rational integration of these three components into a single multifunctional nanocomposite capable of addressing MPs with different surface chemistries and polymer backbones. In particular, the aromatic phenyl groups of polystyrene are expected to interact favorably with the π-rich domains of *g*-C_3_N_4_ and the imidazolate framework of ZIF-67, whereas polyethylene and polypropylene, both nonpolar polyolefins, are expected to be removed mainly through hydrophobic attraction, surface adhesion, and physical entrapment within the porous chitosan-supported framework. The morphology, porosity, crystallinity, and surface chemistry of the synthesized nanocomposite were investigated by XPS, XRD, FTIR, BET, EDX, SEM, and TEM. Its adsorption performance was evaluated in batch mode using different water matrices, and its stability was assessed under harsh pH and salinity conditions. By combining MOF porosity, carbon nitride interfacial functionality, and biopolymer processability, this study introduces a regenerable and environmentally compatible platform for microplastic remediation and provides mechanistic insight into the design of advanced hybrid sorbents for aquatic decontamination.

## 2. Reagents and Methods

### 2.1. Reagents

Analytical-grade reagents were used without modification unless stated otherwise. Cobalt nitrate hexahydrate Co(NO_3_)_2_·6H_2_O, 2-methylimidazole, methanol, acetic acid (purity ≥ 99.5%), epichlorohydrin, chitosan (CS; 200–600 mPa·s, 0.5% in 0.5% acetic acid at 20 °C), sodium hydroxide (NaOH), and hydrochloric acid (HCl, 36%) were purchased from Sigma-Aldrich, located in St. Louis, MO, USA. Graphitic carbon nitride (*g*-C_3_N_4_) powder was supplied by Sinopharm Chemical Reagent Co., Ltd. (Shanghai, China). Deionized water was used throughout all synthesis and washing steps. For the adsorption experiments, commercial polypropylene (PP), polystyrene (PS), and polyethylene (PE) microplastic powders with particle sizes of 20–25 μm were used; the PP and PS powders were sourced from the Arabian Plastic Industrial Company (APICO, Jeddah, Saudi Arabia).

### 2.2. Preparation of ZIF-67/g-C_3_N_4_/CS Nanocomposite

The ZIF-67/*g*-C_3_N_4_/CS nanocomposite was prepared in three sequential steps: (i) solvothermal synthesis of ZIF-67 from cobalt nitrate hexahydrate and 2-methylimidazole in methanol at 120 °C for 12 h; (ii) fabrication of a crosslinked *g*-C_3_N_4_/chitosan matrix by dispersing *g*-C_3_N_4_ in chitosan solution followed by epichlorohydrin crosslinking under alkaline conditions; and (iii) mechanochemical functionalization of the dried *g*-C_3_N_4_/CS powder with ZIF-67 using a stainless-steel ball-mill reactor for 20 min at 30 Hz to obtain the final nanocomposite at a mass ratio of 2:1:2.

*(i)*  *Synthesis of ZIF-67*

ZIF-67 was synthesized through a solvothermal route using cobalt nitrate hexahydrate as the cobalt source and 2-methylimidazole as the organic linker [35,36]. In a typical preparation, 5.82 g of cobalt nitrate hexahydrate Co(NO_3_)_2_·6H_2_O was dissolved in 100 mL of methanol to form solution A. Separately, 13.14 g of 2-methylimidazole was dissolved in 100 mL of methanol to form solution B. Solution B was added slowly to solution A under vigorous magnetic stirring, and the resulting purple mixture was stirred for 30 min at room temperature to ensure complete homogenization. The mixed solution was then transferred into a 250 mL Teflon-lined stainless-steel autoclave and heated at 120 °C for 12 h. After natural cooling to room temperature, the purple precipitate was collected by centrifugation, washed three times with methanol to remove unreacted species, and dried in a vacuum oven at 60 °C for 12 h. The obtained ZIF-67 powder was stored in a desiccator until use. To prepare the final composite at a 2:1:2 mass ratio, 2.0 g of the dried ZIF-67 was reserved for the subsequent ball-milling step.

*(ii)*  *Preparation of ECH-crosslinked g-C_3_N_4_/CS matrix*

A crosslinked *g*-C_3_N_4_/chitosan matrix was prepared using epichlorohydrin (ECH) as the crosslinking agent [37]. Chitosan (2.0 g) was dissolved in 100 mL of 2.0 vol% acetic acid under stirring for 12 h. Separately, *g*-C_3_N_4_ (1.0 g) was dispersed in 50 mL of deionized water and ultrasonicated for 1 h, then added slowly to the chitosan solution and stirred for an additional 4 h. The mixture pH was adjusted to 10.0 with 1.0 M NaOH, followed by dropwise addition of 2.0 mL of ECH. Crosslinking was carried out at 50 °C for 6 h to form a water-stable *g*-C_3_N_4_/CS network while preserving the functional groups of both components. The product was washed repeatedly with deionized water to neutral pH, dried at 60 °C for 24 h, and gently ground to obtain the crosslinked *g*-C_3_N_4_/CS powder at a 1:2 mass ratio.

*(iii)*  *Functionalization of crosslinked g-C_3_N_4_/CS with ZIF-67 by ball milling*

The final ZIF-67/*g*-C_3_N_4_/CS nanocomposite was prepared by mechanochemical functionalization of the crosslinked *g*-C_3_N_4_/CS matrix with ZIF-67 using a ball mill reactor. For this purpose, 2.0 g of the previously prepared ZIF-67 and 3.0 g of the dried *g*-C_3_N_4_/CS powder (containing 1.0 g *g*-C_3_N_4_ and 2.0 g CS) were introduced into a 50 mL zirconia milling jar containing two stainless balls. The powder mixture was milled at 30 Hz for 20 min. This mechanochemical step promotes intimate interfacial contact between the porous ZIF-67 particles and the crosslinked *g*-C_3_N_4_/CS support without the need for additional solvents or harsh thermal treatment. The resulting composite was recovered as a fine purple-gray powder and stored in a desiccator for further characterization and adsorption experiments.

### 2.3. Characterization Techniques

The textural properties of the synthesized nanocomposites, including specific surface area and pore size distribution, were determined from N_2_ adsorption–desorption isotherms using a Quantachrome NOVA 4200e surface area analyzer(Boynton Beach, FL, USA). The concentrations of PP, PE, and PS microplastics in the treated suspensions were measured using a turbidimeter (Hach 2100Q/2100Q, Loveland, CO, USA). The morphology and microstructure of the ZIF-67/CS/*g*-C_3_N_4_ nanocomposite were examined using scanning electron microscopy (SEM; JEOL JSM-6010LV, Akishima, Tokyo, Japan) and high-resolution transmission electron microscopy (TEM; JEOL JEM-2100, Akishima, Tokyo, Japan). The surface elemental composition and chemical states were analyzed using X-ray photoelectron spectroscopy (XPS; Thermo ESCALAB 250Xi, East Grinstead, UK). Crystalline characteristics and phase composition were investigated using a Rigaku D/MAX-2550 diffractometer (Akishima, Tokyo, Japan) with Cu Kα radiation was utilized for the powder X-ray diffraction (XRD) analysis. Functional groups present in the composite were identified using Fourier transform infrared spectroscopy (FTIR; Nicolet 400, Madison, WI, USA) over the wavenumber range of 4000–400 cm^−1^. The surface charge behavior of the material was evaluated through zeta potential analysis using a Zetasizer Nano ZS90 (Malvern, UK) on a 1.0 wt% aqueous suspension. Monitoring of the solution pH was carried out using a calibrated Model 810 pH meter from Fisher Scientific (Waltham, MA, USA), equipped with a combined glass electrode.

### 2.4. PS/PE/PP-Mps Batch Sorption Study

For the microplastic sorption experiments, 100 mg of the synthesized ZIF-67/*g*-C_3_N_4_/CS nanocomposite was dispersed in 10 mL of microplastic suspension containing PP, PE, or PS. The mixtures were agitated in a thermostatic shaker at 250 rpm and 25 °C for 40 min to ensure sufficient contact between the adsorbent and the suspended microplastic particles. After equilibration, the suspensions were filtered, and the residual microplastic content in the filtrate was determined by turbidity measurement. Turbidimetry was selected as the analytical method because it provided higher sensitivity for suspended microplastic quantification under the present conditions than direct absorbance measurements. Calibration curves were established separately for each microplastic type, and all showed excellent linearity with *R*^2^ values above 0.995. All sorption experiments were conducted in triplicate, and the reported data represents the mean values of the independent measurements.

Consecutive sorption–desorption runs were conducted to assess the reusability of the ZIF-67/*g*-C_3_N_4_/CS nanocomposite. The exhausted adsorbent was collected at the end of each cycle and dried for a 12 h period before the next run. Desorption was then carried out by immersing the used nanocomposite in 20 mL of ethanol and shaking the suspension at 250 rpm and 25 °C for 40 min. Following desorption, the regenerated material was separated and dried before being utilized in the next sorption run under identical conditions. The adsorption efficiency before and after regeneration was compared to assess the stability and recyclability of the nanocomposite over repeated use. PS/PE/PP-MPs removal efficiency and adsorption capacity were calculated using Equations (1) and (2), respectively:

(1) $$\%\;R=\;\;\frac{C_{0}-\;C_{t}\;}{c_{0}}\;\;\times \;\;100$$

(2) $$\%\;q_{t}=\;\frac{{(C}_{0}-C_{t})\;V\;}{m}$$

where *C*_0_ and *C*_*t*_ (mg L^−1^) represent the initial microplastic concentration and the concentration remaining at time t, respectively; *V* (L) is the solution volume; and *m* (*g*) is the mass of the nanocomposite used in the adsorption experiment. All adsorption measurements were carried out in triplicate, and the reported values correspond to the average of the independent experiments. The analytical performance of the method was assessed in terms of precision and accuracy through replicate determinations, using recovery values and relative standard deviation (RSD) as evaluation criteria.

The adsorption behavior of the ZIF-67/*g*-C_3_N_4_/CS nanocomposite toward PP, PE, and PS microplastics was systematically evaluated as a function of solution pH, salinity, and coexisting ions using suspensions with an initial microplastic concentration of 15 mg L^−1^. For the pH study, the solution pH was adjusted over the range of 2.0–10.0 using 0.1 M of HCl or NaOH. The effect of competing ions was investigated in the presence of common inorganic species, including the anions Cl^−^, NO_3_^−^, and SO_4_^2−^, and the cations K^+^, Na^+^, Mg^2+^, and Ca^2+^, each introduced at a 20 mM concentration. Salinity tolerance was assessed using NaCl concentrations from 5 to 500 mM while maintaining the solution at pH 6.2. These experiments were performed to examine the selectivity of the adsorbent and to assess its stability under conditions representative of natural and engineered water systems.

The reusability of the nanocomposite was examined through adsorption–desorption cycling under equilibrium conditions. In each cycle, adsorption was carried out at 200 rpm and 25 °C for 40 min, followed by regeneration of the spent material using ethanol as the desorbing medium. The regenerated nanocomposite was then recycled in subsequent runs under the same operating conditions. This procedure was repeated for five consecutive cycles, and all measurements were conducted in triplicate to evaluate the operational stability, regeneration efficiency, and durability of the material during repeated microplastic capture.

To assess the practical applicability of the developed nanocomposite, adsorption experiments were further performed using a range of real water and wastewater matrices, including Milli-Q water, seawater, river water, tap water, and two domestic wastewater samples. Seawater was sampled along Egypt’s Mediterranean coast from August to October 2025, while freshwater samples were gathered from the Nile River, and tap water was sourced directly from a laboratory tap at Alexandria University, Egypt. Before experimental use, each environmental water sample was clarified using 0.22 μm membrane filters and maintained at a temperature of 4 °C. Table 1 outlines the primary physicochemical characteristics of these water matrices, such as pH, total dissolved solids (TDS), and predominant ionic compositions. Domestic wastewater, tap water, and river water samples were analyzed directly, whereas seawater samples were diluted 1:1000 before ion analysis because of their high salinity. This set of experiments was designed to determine whether the adsorption performance observed in model systems could be maintained under the chemical complexity of realistic environmental matrices.

## 3. Results and Discussion

### 3.1. Characterization of the ZIF-67/g-C_3_N_4_/CS

**Figure 1:**
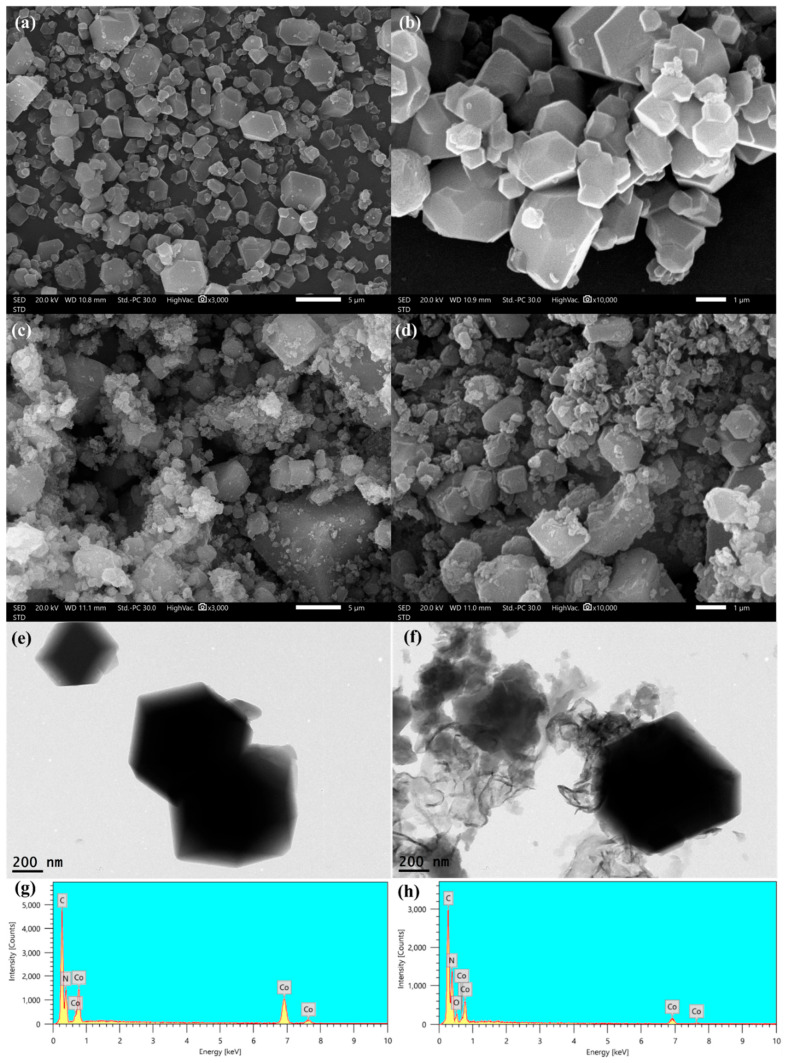
Morphological and compositional characterization of synthesized materials. SEM images of (**a**,**b**) ZIF-67 and (**c**,**d**) ZIF-67/CS/*g*-C_3_N_4_ nanocomposite at different magnifications; (**e**,**f**) TEM images of (**e**) ZIF-67 and (**f**) ZIF-67/CS/*g*-C_3_N_4_; (**g**,**h**) EDX spectra of (**g**) ZIF-67 and (**h**) ZIF-67/CS/*g*-C_3_N_4_.

Scanning electron microscopy (SEM) and transmission electron microscopy (TEM) were employed to examine the surface topography and microstructural traits of the fabricated samples, while elemental composition was confirmed by EDX. The SEM images of ZIF-67 (Figure 1a,b) reveal well-defined polyhedral crystals with a uniform rhombic dodecahedral morphology, characteristic of highly crystalline ZIF-67 structures [38]. These particles are relatively homogeneous in size with smooth surfaces, indicating successful formation of the pristine framework. In contrast, the ZIF-67/C_3_N_4_/CS nanocomposite (Figure 1c,d) exhibits a markedly altered morphology, where the ZIF-67 crystals are embedded within an irregular, rough, and aggregated matrix. The presence of *g*-C_3_N_4_ sheets and cross-linked chitosan leads to partial coverage and clustering of the ZIF-67 particles, suggesting strong interfacial interactions and effective integration of the components [39,40]. TEM analysis further supports these observations; pristine ZIF-67 (Figure 1e) shows distinct, dense polyhedral particles with sharp edges, whereas the composite (Figure 1f) displays ZIF-67 particles dispersed within a semi-transparent, wrinkled matrix attributed to *g*-C_3_N_4_ and polymeric chitosan, confirming successful hybridization at the nanoscale. Additionally, EDX spectra (Figure 1g,h) verify the elemental composition, where ZIF-67 shows dominant peaks of Co along with C and N, while the composite demonstrates an increased contribution of C and N elements with comparatively reduced Co intensity, consistent with the incorporation of *g*-C_3_N_4_ and chitosan. The absence of impurity peaks further confirms the purity of the synthesized materials.

The N_2_ adsorption–desorption isotherm of the ZIF-67/*g*-C_3_N_4_/CS nanocomposite, measured at 77.40 K, exhibited a typical type IV isotherm with a pronounced hysteresis loop (Figure S1), indicating its mesoporous structure. The material showed a BET-specific surface area of 761.5 m^2^ g^−1^, an average pore diameter of 11.46 nm, and a total pore volume of 0.0813 cm^3^ g^−1^. The volume of nitrogen adsorbed at standard temperature and pressure (STP), corresponding to monolayer formation on the nanocomposite surface, was also determined. Collectively, these results demonstrate the successful fabrication of the ZIF-67/*g*-C_3_N_4_/CS nanocomposite with modified surface morphology and enhanced structural complexity, which are expected to provide abundant active sites and improved adsorption performance toward microplastics.

**Figure 2:**
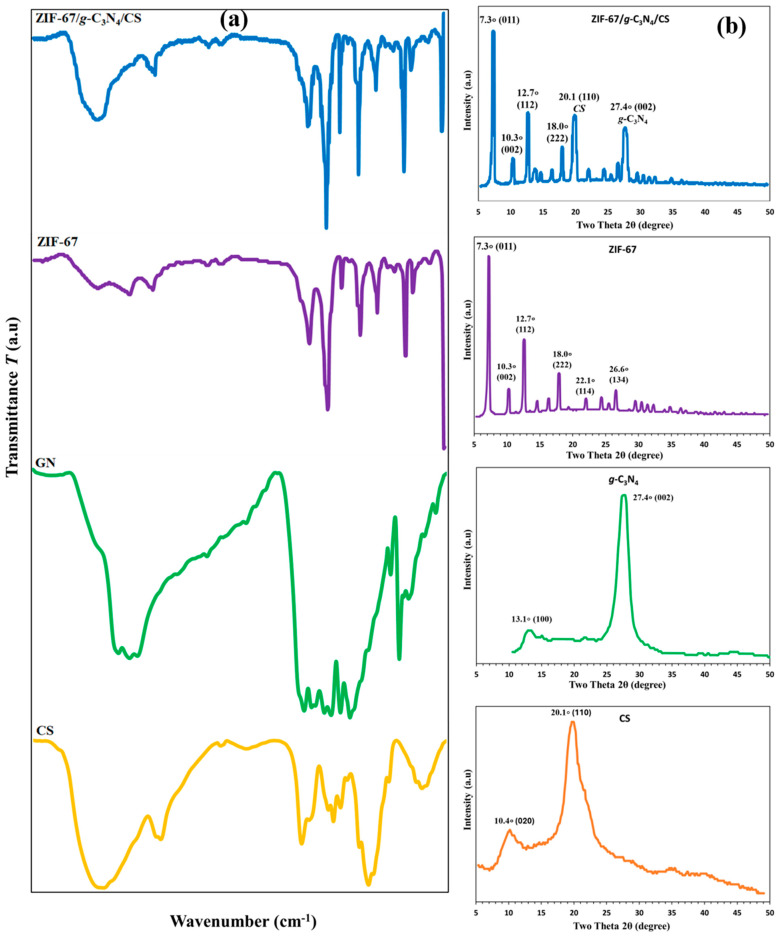
FTIR (**a**) and XRD (**b**) images of CS, *g*-C_3_N_4_, ZIF-67 and ZIF-67/*g*-C_3_N_4_/CS.

FTIR analysis was employed to investigate the surface functional groups and confirm the structural integration within the synthesized materials. Figure 2a displays the FTIR spectra of pristine CS, *g*-C_3_N_4_, pure ZIF-67, and the ternary ZIF-67/*g*-C_3_N_4_/CS nanocomposite. The spectrum of pristine CS exhibits a broad, characteristic absorption band centered at approximately 3400 cm^−1^, corresponding to the overlapping stretching vibrations of hydroxyl (-OH) and 1° amine (-NH_2_) groups [39,40]. The peaks observed at 2920 cm^−1^ and 2875 cm^−1^ are attributed to the asymmetric and symmetric C-H stretching vibrations of the aliphatic methylene blocks. Additionally, characteristic bands appearing at 1655 cm^−1^ and 1590 cm^−1^ correspond to the C=O stretching (Amide I) and N-H bending (Amide II) vibrations, respectively, while the intense peak at 1030 cm^−1^ signifies the C-O-C skeletal stretching of the glucan ring. For the bulk *g*-C_3_N_4_ sample, the prominent sharp peak at 810 cm^−1^ is assigned to the out-of-plane bending vibration of the heptazine (tri-s-triazine) rings. The series of intense stretching bands within the 1200–1650 cm^−1^ region are characteristically associated with the aromatic C-N and C=N stretching vibrations of the conjugated heterocycles. The broad absorption band spanning 3000–3300 cm^−1^ originates from the stretching modes of terminal amino groups (-NH or NH_2_ units) at the layer edges. In the pristine ZIF-67 spectrum, distinct absorption peaks are resolved in the fingerprint region. The bands located at 1300–1500 cm^−1^ belong to the entire imidazole ring stretching vibrations. The peaks at 1140 cm^−1^ and 995 cm^−1^ are associated with the in-plane and out-of-plane bending modes of the imidazole ring, respectively. Crucially, the sharp absorption peak at 425 cm^−1^ confirms the formation of the tetrahedral coordination between the cobalt centers and nitrogen atoms (Co-N stretching). Following the assembly of the ternary ZIF-67/*g*-C_3_N_4_/CS nanocomposite, all principal characteristic footprints of the individual building blocks are preserved, verifying successful hybridization. Notably, the broad -OH/-NH absorption envelope near 3400 cm^−1^ displays a distinct shift and broadening, indicating the formation of robust intermolecular hydrogen-bonding networks among CS, GN, and ZIF-67 [39,40]. Furthermore, the persistence of both the Co-N vibration (425 cm^−1^) and the heptazine ring deformation (810 cm^−1^) confirms that the crystal frameworks of ZIF-67 and *g*-C_3_N_4_ remain intact during the composite fabrication process. This abundant matrix of nitrogen- and oxygen-containing functional groups (-NH, -OH, and imidazole rings) provides a highly favorable surface environment for trapping microplastics (PE, PP, and PS) through electrostatic interactions, hydrogen bonding, and π-π interactions [41,42].

The crystalline architecture and phase purity of the prepared adsorbents were evaluated via XRD profiling, as illustrated in Figure 2b. The XRD pattern of pristine CS reveals a typical semi-crystalline profile with a broad prominent reflection at 2θ = 20.1° and a weaker shoulder around 2θ = 10.4°, corresponding to the (020) and (110) crystallographic planes of the amorphous–crystalline regions of CS. For the *g*-C_3_N_4_ sample, two distinct characteristic peaks are resolved. The primary, highly intense diffraction peak located at 2θ = 27.4° indexes to the (002) plane of graphitic-like materials, representing the characteristic interlayer aromatic stacking distance (d = 0.326 nm). A secondary minor reflection at 2θ = 13.1° corresponds to the (100) plane, which is associated with the in-plane structural repeat units of the tri-s-triazine rings. The diffractogram of pure ZIF-67 displays a series of very sharp, well-defined Bragg reflections at 2θ = 7.3°, 10.3°, 12.7°, 14.7°, 16.5°, 18.0°, 22.1°, 24.5°, 26.6°, and 29.6°. These peaks match perfectly with the simulated sodalite (SOD) topology of ZIF-67 reported in the literature, confirming successful synthesis and excellent long-range crystalline order [38]. In the XRD pattern of the ternary ZIF-67/*g*-C_3_N_4_/CS nanocomposite, the highly crystalline diffraction peaks of ZIF-67 remain dominant, demonstrating that the in situ growth of the metal–organic framework occurred successfully without disrupting its structural integrity or topology. Concurrently, the broad crystalline feature of CS (20.1°) and the characteristic interlayer stacking peak of *g*-C_3_N_4_ (27.4°) are incorporated into the composite pattern [39,40]. The slight reduction in the relative peak intensities of ZIF-67 within the composite profile is attributed to the dilution effect and the shielding effects exerted by the wrapping of the amorphous/semi-crystalline chitosan chains and the insertion of *g*-C_3_N_4_ sheets. No secondary phases or uncoordinated cobalt oxide impurities were observed, establishing the high purity and structural stability of the ternary composite designed for microplastic remediation.

**Figure 3:**
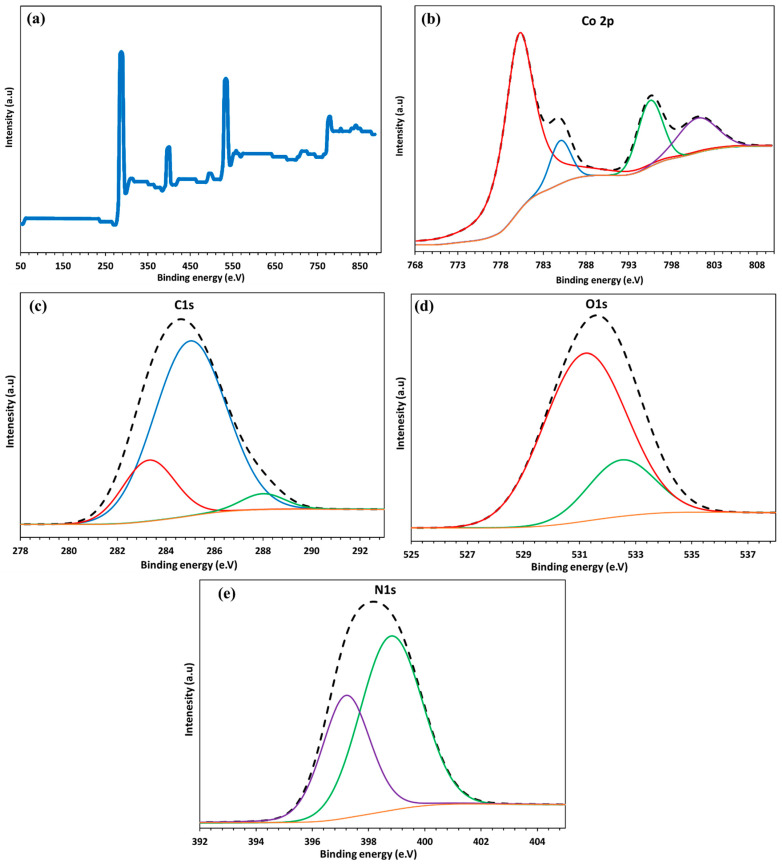
Full spectrum (**a**) of ZIF-67/*g*-C_3_N_4_/CS nanocomposite and high-resolution XPS images (**b**–**e**) of Co2p, C1s, O1s, and N1s.

To elucidate the surface elemental composition and chemical states of the synthesized ZIF-67/*g*-C_3_N_4_/CS nanocomposite, XPS analysis was conducted. The XPS wide-scan survey spectrum (Figure 3a) confirms the coexistence of all constituent elements within the ternary composite framework. Distinct characteristic photoelectron lines are observed corresponding to Cobalt (Co 2p), oxygen (O 1s), nitrogen (N 1s), and carbon (C 1s), verifying the successful integration of ZIF-67, *g*-C_3_N_4_, and CS [39,40]. No alternative elemental impurities were detected, underscoring the high purity of the synthesized nanosorbent. The high-resolution core-level spectrum of Co 2p (Figure 3b) exhibits a complex spin–orbit doublet characteristic of the cobalt ions within the ZIF-67 framework. The spectrum is deconvoluted into two primary peaks situated at approximately 780.4 eV and 796.1 eV, assigned to Co 2p1/2 and Co 2p3/2 states, respectively. The spin–orbit splitting energy Δ*E* of 15.7 eV is indicative of a Co(II) oxidation state. Furthermore, the presence of distinct shake-up satellite peaks located at higher binding energies 785.2 eV and 801.5 eV confirm the high-spin divalent nature of cobalt Co(II) coordinated within the imidazole links. The deconvoluted C1s core-level spectrum (Figure 3c) resolves into three primary chemical environments. The dominant peak centered at 284.8 eV is ascribed to the adventitious or graphitic carbon C–C/C=C bonds) present in the chitosan backbone and the carbon networks. The peak located around 283.4 eV represents the C–N coordinated bonds, signaling the interfacial linkage between chitosan and the *g*-C_3_N_4_ matrix [39,40]. Additionally, a minor peak at 288.0 eV corresponds to the N–C=N sp^2^-bonded carbon species originating from the triazine units of *g*-C_3_N_4_. The high-resolution O1s profile (Figure 3d) provides insight into the oxygenated functionalities linked to the chitosan component [40]. Deconvolution yields two distinct components: a primary peak at 531.2 eV associated with the surface hydroxyl groups (–OH) or adsorbed water molecules, and a shoulder peak at 532.6 eV attributed to C–O ether or alcohol bonds within the pyranose rings of chitosan. These oxygen-containing moieties play a vital role in providing active binding sites for microplastic interactions through hydrogen bonding. The N 1s core-level region (Figure 3e) can be resolved into two major components, reflecting the rich nitrogen chemistry of both ZIF-67 and *g*-C_3_N_4_. The peak at 397.3 eV is assigned to the pyridinic-like nitrogen (C–N=C) within the triazine rings of *g*-C_3_N_4_ and the imidazole ring of ZIF-67 [41,42]. The prominent peak at 398.9 eV is assigned to the tertiary nitrogen [N–(C)_3_] connections within the *g*-C_3_N_4_ network, potentially overlapping with the amino groups (–NH_2_) of chitosan [39,40]. The slight binding energy shifts observed in these core levels compared to pristine precursors confirm strong electronic interactions and interfacial coupling among ZIF-67, *g*-C_3_N_4_, and chitosan. This synergistic network enhances surface area and active site density, facilitating the efficient adsorption and removal of microplastics (PE, PP, and PS) via electrostatic attractions, π-π stacking, and hydrophobic interactions [41,42].

### 3.2. Impact of pH, Salinity, and Co-Existing Cations/Anions

**Figure 4:**
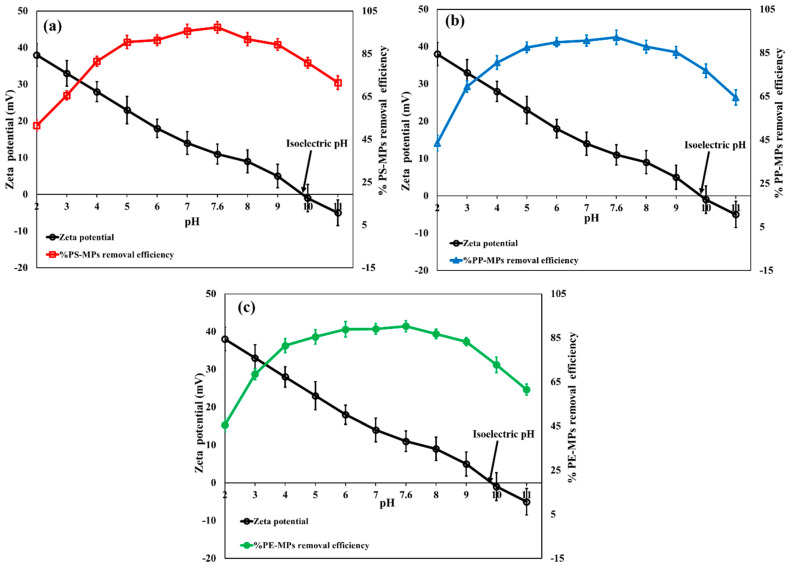
Variation in the zeta potential of the ZIF-67/*g*-C_3_N_4_/CS nanocomposite with solution pH and the associated changes in the microplastics percentage removal of PS (**a**), PP (**b**), and PE (**c**).

Solution pH is a critical variable because it governs the surface charge of the ZIF-67/*g*-C_3_N_4_/CS nanocomposite, the interfacial behavior of suspended microplastics, and the balance between electrostatic and non-electrostatic adsorption forces. As shown in Figure 4a–c, the zeta potential of the nanocomposite decreased progressively with increasing pH, changing from strongly positive values under acidic conditions to near-neutral and then slightly negative values in alkaline media. The surface remained positively charged over a broad pH range and approached charge neutrality only at high pH, indicating that the chitosan-rich hybrid framework retained substantial cationic character well above mildly acidic conditions. In parallel, the removal efficiencies of PS, PP, and PE increased from acidic pH to near-neutral conditions, reached a maximum at pH 7.6, and remained comparatively stable within the pH range 5–9, after which a clear decline was observed in strongly alkaline media.

The adsorption behavior in this pH window cannot be explained by electrostatic attraction alone. If electrostatics were the dominant mechanism, the highest removal would be expected at the lowest pH, where the zeta potential is most positive. Instead, the best performance occurred at pH 7.6, where the nanocomposite still carried a moderately positive surface charge, but the adsorption interface was likely more favorable for particle approach, surface adhesion, and diffusion into the porous hybrid structure. This indicates that the removal of PP, PE, and PS microplastics by ZIF-67/*g*-C_3_N_4_/CS is governed by a combination of mechanisms, including hydrophobic attraction, van der Waals interactions, physical trapping, hydrogen-bond-assisted interfacial effects, and, for PS, π-related interactions, in addition to any contribution from electrostatic forces [43,44]. The comparable removal efficiencies observed between pH 5 and 9 therefore suggest that the adsorption process is robust over a broad environmentally relevant range and is sustained by the multifunctional surface chemistry of the nanocomposite rather than by surface charge alone.

The lower performance observed in strongly acidic media is most reasonably attributed to the unfavorable adsorption environment created by excess proton activity, which can alter the hydration layer around the composite surface, influence particle dispersion, and reduce the effectiveness of interfacial adhesion despite the high positive charge of the adsorbent [45]. By contrast, the decline in strongly basic media is consistent with the progressive decrease in positive surface charge, eventual approach to near-zero or negative zeta potential, and corresponding reduction in favorable interfacial interactions between the nanocomposite and the suspended microplastics. Under these conditions, electrostatic screening and changes in colloidal stability become more pronounced, leading to weaker overall capture efficiency. Thus, the maximum performance at pH 7.6 reflects an optimum balance between surface charge, particle accessibility, and non-electrostatic adsorption forces, while the high efficiencies maintained from pH 5 to 9 confirm the broad operational tolerance of the developed hybrid material.

**Figure 5:**
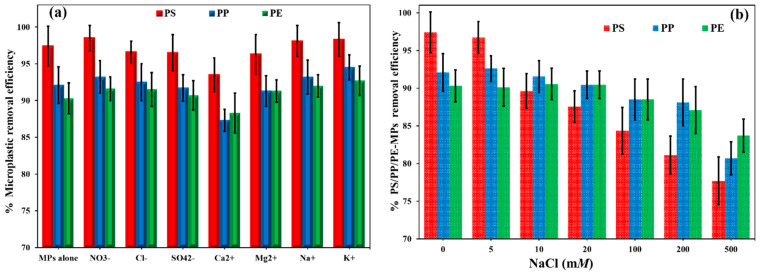
Effects of competing ions (**a**) and salinity (**b**) on the percentage of microplastics removal of PP, PE, and PS by the ZIF-67/*g*-C_3_N_4_/CS nanocomposite.

As shown in Figure 5a, coexisting inorganic ions affected the adsorption of PP, PE, and PS microplastics by the ZIF-67/*g*-C_3_N_4_/CS nanocomposite, although the magnitude of the effect depended strongly on ion valence and identity. Under the optimum adsorption condition (pH 7.6), the nanocomposite exhibited its highest removal efficiencies in the absence of added competing ions, reaching approximately 97–98% for PS, 92–93% for PP, and 89–90% for PE. In the presence of Ca^2+^, these values decreased to about 88–89% for PS, 79–80% for PP, and 74–75% for PE, representing the strongest inhibition among the tested ions. A noticeable but less severe decline was also observed with Mg^2+^, for which the removal efficiencies were reduced to approximately 90–91% for PS, 82–84% for PP, and 78–80% for PE. By contrast, the monovalent cations Na^+^ and K^+^ caused more limited reductions, with adsorption remaining above roughly 90% for PS, 84–87% for PP, and 80–83% for PE.

This trend clearly indicates that divalent cations interfere more strongly than monovalent cations, primarily because they compress the electrical double layer more effectively and promote aggregation of suspended microplastic particles, thereby limiting access of the particles to the active adsorption domains of the nanocomposite [46]. In addition, divalent ions can interact more strongly with the oxygen-containing functionalities present in the hybrid matrix, which may further alter the adsorption interface. Among the cations examined, Ca^2+^ exerted the most pronounced inhibitory effect, decreasing the removal efficiency by nearly 9–10 percentage points for PS, 12–13 percentage points for PP, and 14–15 percentage points for PE relative to the ion-free system. This behavior is consistent with its stronger charge-screening effect and greater ability to induce colloidal destabilization and particle bridging [47,48].

The influence of anions was comparatively less pronounced. In the presence of SO_4_^2−^, NO_3_^−^, and Cl^−^, the removal efficiencies remained relatively high, generally within the ranges of 90–95% for PS, 82–88% for PP, and 79–84% for PE. Although sulfate produced a somewhat larger decrease than nitrate and chloride, the overall impact of anions was still smaller than that of the divalent cations. This suggests that adsorption was governed predominantly by cation-mediated changes in colloidal stability rather than by direct competition from anions at the adsorbent interface. Collectively, these results demonstrate that the ZIF-67/*g*-C_3_N_4_/CS nanocomposite retains strong adsorption capability under chemically complex conditions, but its performance is most sensitive to multivalent cations, especially Ca^2+^, which significantly reduces favorable contact between suspended microplastics and the multifunctional adsorbent surface.

The influence of ionic strength on the adsorption of PS, PP, and PE microplastics by the ZIF-67/*g*-C_3_N_4_/CS nanocomposite is presented in Figure 5b. These experiments were performed at an optimum pH of 7.6, where the nanocomposite exhibits its maximum intrinsic performance. Under low-salinity conditions, the nanocomposite showed the highest removal efficiencies, with approximate values of 90–97% for PS, 90–93% for PP, and 90–91% for PE. Thus, at low ionic strength, the adsorption followed the order PS > PP > PE, which is consistent with the stronger affinity of PS for the hybrid surface. This higher affinity is reasonably attributed to the aromatic structure of PS, which can interact more favorably with the π-conjugated domains of *g*-C_3_N_4_ and the imidazolate-containing framework of ZIF-67, in addition to hydrophobic and dispersive interactions. By contrast, PP and PE, being non-aromatic polyolefins, are retained mainly through hydrophobic attraction, van der Waals interactions, and physical trapping within the porous chitosan-supported architecture.

As the NaCl concentration increased from freshwater-like to seawater-like levels, the removal efficiency decreased progressively for all three microplastics, although the extent of suppression was different in each case. The strongest salinity effect was observed for PS, whereas PE showed the greatest tolerance to ionic strength. Accordingly, the performance order changed at high salinity from PS > PP > PE to approximately PE ≥ PP > PS. A statistically significant decrease in adsorption was evident at higher ionic strength, confirming that salinity exerts a measurable matrix effect on the adsorption process.

This salinity-dependent behavior can be explained by electrical double-layer compression, electrostatic screening, and salt-induced microplastic aggregation, all of which reduce the accessibility of particle surfaces to the active adsorption domains of the nanocomposite. At elevated electrolyte concentrations, the colloidal stability of suspended microplastics decreases, promoting particle–particle association and limiting favorable contact with the adsorbent surface. This effect is especially pronounced for PS, whose adsorption at low salinity benefits strongly from interfacial interactions that become more easily disrupted under highly saline conditions. Nevertheless, adsorption was not completely suppressed even at the highest salinity tested, indicating that non-electrostatic interactions remained operative. The residual uptake is therefore most likely sustained by hydrophobic forces and van der Waals attraction, which become relatively more important when electrostatic contributions are weakened [45]. Overall, these findings show that the high adsorption efficiency observed at pH 7.6 originates from a favorable balance of interfacial interactions, whereas increasing salinity progressively limits capture efficiency by altering suspension stability and particle accessibility rather than by eliminating adsorption altogether.

### 3.3. Reusability of ZIF-67/g-C_3_N_4_/CS

**Figure 6:**
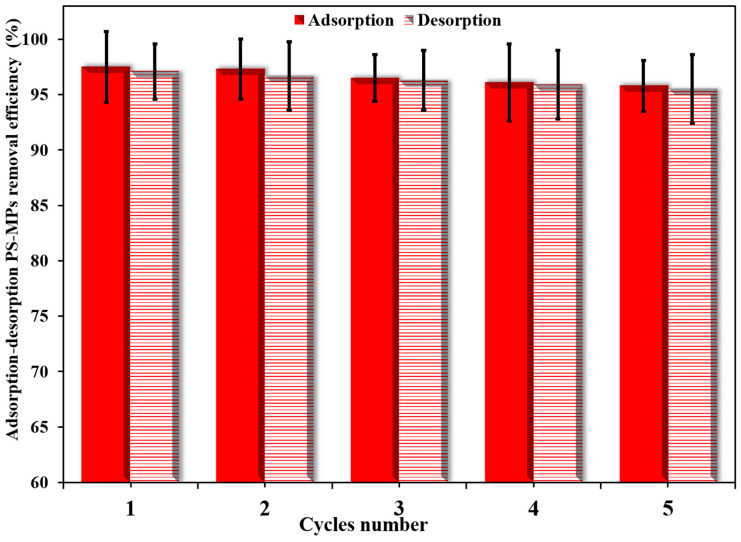
Reusability of the ZIF-67/*g*-C_3_N_4_/CS nanocomposite and its removal efficiency toward PS microplastics under the optimum adsorption conditions (pH 7.6, contact time 25 min, and temperature 25.0 °C).

Reusability is a key criterion in assessing the practical and economic suitability of adsorbents for water treatment applications. To examine the regeneration performance of the ZIF-67/*g*-C_3_N_4_/CS nanocomposite toward PS microplastics, repeated adsorption–desorption experiments were carried out under equilibrium conditions. In each cycle, adsorption was followed by regeneration of the spent material using ethanol as the desorbing medium, after which the recovered adsorbent was reused in the subsequent run. As shown in Figure 6, the nanocomposite preserved consistently high PS removal efficiency throughout the recycling study, maintaining an average performance above 95.1% over five consecutive cycles. Although a slight decrease relative to the initial cycle was observed, the decline remained limited, indicating that the active adsorption domains of the material were largely retained during repeated use.

The minor loss in performance is reasonably attributed to partial occupation or shielding of adsorption sites by strongly retained microplastic particles, which may not be completely removed during the desorption step [49]. Nevertheless, the sustained adsorption efficiency demonstrates that the hybrid structure of ZIF-67/*g*-C_3_N_4_/CS possesses good structural stability and regeneration tolerance. These findings confirm that the fabricated nanocomposite can be reused multiple times without significant deterioration in adsorption performance, underscoring its promise as a durable and practically deployable adsorbent for microplastic remediation.

### 3.4. Kinetic Study and Impact of Time

**Figure 7:**
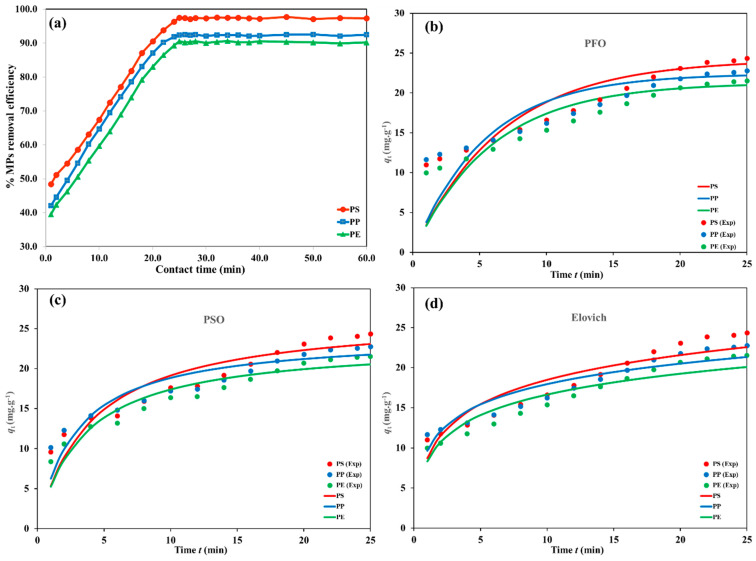
Impact of contact time (**a**), PFO (**b**), PSO (**c**) and Elovich (**d**) kinetic models for the PS/PP/PE-MPs adsorptive removal by ZIF-67/*g*-C_3_N_4_/CS nanocomposite.

The adsorption kinetics of PS, PP, and PE microplastics onto the ZIF-67/*g*-C_3_N_4_/CS nanocomposite exhibit a rapid initial uptake followed by a gradual approach to equilibrium, as clearly observed in the experimental profiles (Figure 7a). For all three microplastics, a significant fraction of adsorption occurs within the first 10–15 min, after which the uptake rate decreases and a plateau is reached at approximately 20–25 min, indicating the progressive occupation of available active sites. The equilibrium adsorption capacities derived from the experimental data follow the order PS (≈24.4 mg g^−1^) > PP (≈22.8 mg g^−1^) > PE (≈21.6 mg g^−1^), suggesting a higher affinity of the composite surface toward PS, which can be attributed to stronger π–π interactions between the aromatic structure of PS and the conjugated domains of ZIF-67 and *g*-C_3_N_4_, in addition to hydrogen bonding and electrostatic contributions from the chitosan matrix.

The nonlinear kinetic fitting reveals that the pseudo-second-order (PSO) model (Table 2) provides the best description of the adsorption process for all microplastics, as evidenced by the highest *R*^2^ values and the lowest RMSE compared to the PFO and Elovich models (Figure 7b–d). Furthermore, the calculated equilibrium capacities (*q*_e,calc_) from the PSO model closely match the experimental values, reinforcing the reliability of this model. In contrast, the PFO model shows noticeable deviations, particularly at longer contact times, where it underestimates the adsorption capacity, indicating that it is less capable of capturing the entire kinetic profile. A PSO fit is described only as an empirical indication that the adsorption rate depends on the availability and progressive occupation of adsorption domains. For PP and PE, the proposed uptake is attributed primarily to hydrophobic adhesion, van der Waals forces, and physical trapping rather than electrostatic attraction or chemical bonding.

Additionally, the Elovich kinetic model demonstrated a satisfactory fit to the empirical results, reflecting the heterogeneous nature of the adsorbent surface. The relatively high initial adsorption rates (*α*) indicate a strong driving force for adsorption at the early stage, while the Elovich constant (*β*), related to surface coverage and activation energy, suggests a gradual decrease in adsorption rate as the surface becomes increasingly occupied. However, the slightly lower correlation coefficients and higher fitting errors compared to the PSO model indicate that, although surface heterogeneity is present, it is not the dominant controlling factor.

Overall, the combined kinetic analysis indicates that adsorption of microplastics onto ZIF-67/*g*-C_3_N_4_/CS is a rapid and efficient process dominated by different mechanisms with contributions from multiple interactions, including π–π stacking, hydrogen bonding, and electrostatic attraction. The consistently higher adsorption capacity and affinity observed for PS compared to PP and PE further highlight the importance of molecular structure in governing adsorption behavior, with aromatic-rich polymers exhibiting stronger interactions with the engineered composite surface.

### 3.5. PS/PP/PE-MPs Sorption Isotherms

The equilibrium adsorption data for PS, PP, and PE were fitted using the nonlinear Langmuir and Freundlich models over the investigated concentration range of 5–50 mg L^−1^ (Table 3). For all three microplastics, the Langmuir model provided the better statistical description, as indicated by its higher coefficients of determination and lower RMSE values compared with the Freundlich model. The fitted limiting-capacity parameters (*q*_m_) were 97.69, 94.86, and 93.67 mg g^−1^ for PS, PP, and PE, respectively. In addition, the calculated separation factors (*R*_L_) were between 0 and 1, indicating favorable adsorption under the tested conditions.

The better Langmuir fit should not, however, be interpreted as direct evidence that the structurally heterogeneous microplastic particles formed a uniform monolayer on an ideally homogeneous surface. In the present system, *q*_m_ is more appropriately regarded as an empirical limiting-capacity parameter defined by the fitted model and constrained by the experimental concentration range. Microplastic particles can differ in shape, surface condition, orientation, and contact geometry, and these variations may produce several interfacial configurations even when the Langmuir equation gives the strongest statistical fit. Parameter uncertainties and 95% confidence intervals were therefore included to provide a more transparent assessment of the fitted values.

The Freundlich model also described the data reasonably well, with (*n* > 1) for all three polymers, confirming favorable adsorption on an energetically heterogeneous composite surface. Nevertheless, its lower *R*^2^ values and higher RMSE values indicate that it was less suitable than the Langmuir model for representing the equilibrium data within the studied range. Thus, model selection was based on comparative fitting performance rather than on a literal assumption of monolayer adsorption. The slightly higher affinity observed for PS is consistent with additional interactions between its aromatic phenyl groups and the π-conjugated domains of *g*-C_3_N_4_ and ZIF-67. In contrast, the adsorption of PP and PE is more reasonably attributed to hydrophobic adhesion, van der Waals forces, and physical retention within the rough and porous composite structure.

### 3.6. Synergistic Roles of CS, g-C_3_N_4_, and ZIF-67 in PP/PE/PS Microplastic Adsorption by ZIF-67/g-C_3_N_4_/CS and Plausible Adsorption Mechanism

To elucidate the contribution of each constituent to the adsorption performance of the fabricated ZIF-67/*g*-C_3_N_4_/CS nanocomposite, comparative experiments were conducted using the individual components and the assembled hybrid material. These findings explicitly demonstrate that the enhanced performance of the ternary nanocomposite stems from a synergistic interaction between CS, *g*-C_3_N_4_, and ZIF-67, rather than the individual contributions of any single component. Among the single components, pristine *g*-C_3_N_4_ displayed the weakest overall adsorption performance, with removal efficiencies of 80.5 ± 2.7% for PS, 62.7 ± 2.5% for PP, and 58.4 ± 3.1% for PE. This behavior is consistent with the structural characteristics of *g*-C_3_N_4_, which offers a layered π-conjugated surface and nitrogen-rich domains that can promote interfacial contact, especially with aromatic polymers such as PS, but provides limited affinity toward non-aromatic polyolefins in the absence of additional binding domains or a highly accessible porous framework.

The incorporation of chitosan improved the adsorption performance, yielding removal efficiencies of 73.8 ± 2.1% for PS, 70.3 ± 2.3% for PP, and 64.1 ± 2.7% for PE. The contribution of chitosan should be interpreted primarily in terms of its hydrophilic polymeric network, amino- and hydroxyl-rich surface chemistry, and strong film-forming capacity, rather than as a highly active adsorbent on its own. Chitosan can enhance particle capture by improving wettability, facilitating particle–surface contact, and providing a flexible matrix that promotes adhesion and localized interfacial interactions. At suitable pH, partial protonation of amino groups may also contribute to the capture of negatively charged or oxidized microplastic surfaces, although this effect depends on the surface aging state of the polymer particles. More generally, chitosan acts as a stabilizing and contact-promoting phase that improves accessibility of the other functional components.

ZIF-67 showed higher adsorption performance than the individual CS and *g*-C_3_N_4_ phases, reaching 86.1 ± 2.8% for PS, 76.1 ± 1.7% for PP, and 70.2 ± 2.1% for PE. This enhanced performance can be attributed to its high specific surface area, porous framework, and chemically heterogeneous imidazolate-rich surface, all of which favor microplastic capture through surface adhesion, pore-associated trapping, and dispersive interfacial interactions. For particles such as PS, which contain aromatic phenyl groups, the imidazolate linkers of ZIF-67 provide a more favorable interfacial environment than would be expected for non-aromatic polymers. However, ZIF-67 alone still lacks the hierarchical interfacial organization required for maximal contact efficiency and broad-spectrum capture of chemically distinct microplastics.

When the three components were integrated into a single hybrid architecture, the adsorption efficiency increased markedly to 97.5 ± 3.2% for PS, 92.2 ± 2.4% for PP, and 90.3 ± 2.5% for PE. These findings validate a significant complementary interaction occurring within the ternary nanocomposite. The excellent performance of the composite is not simply a cumulative effect of the three constituents but rather reflects the formation of a multifunctional interface in which the role of each moiety becomes amplified by the presence of the others. Chitosan provides structural cohesion and hydrophilic processability, *g*-C_3_N_4_ contributes π-rich layered domains and additional surface roughness, and ZIF-67 supplies high porosity and abundant accessible adsorption sites. Together, these features generate a surface that is chemically diverse, physically heterogeneous, and highly favorable for microplastic capture.

The adsorption mechanism of PP, PE, and PS on the ZIF-67/*g*-C_3_N_4_/CS nanocomposite is best understood as a combination of hydrophobic attraction, van der Waals interactions, surface adhesion, pore-assisted trapping, hydrogen-bond-assisted interfacial interactions, and, in the case of PS, π-related interactions. Because PP and PE are nonpolar polyolefins lacking aromatic rings and strong polar functionalities, their uptake is governed predominantly by hydrophobic interactions and dispersive van der Waals forces, together with physical retention within the porous and roughened composite surface. Their relatively lower removal efficiency compared with PS is therefore consistent with their chemically inert structure and limited capacity for specific interfacial interactions.

By contrast, PS contains pendant aromatic phenyl groups, which provide additional opportunities for interaction with the π-conjugated planes of *g*-C_3_N_4_ and the aromatic imidazolate linkers of ZIF-67. Accordingly, the stronger adsorption of PS can be reasonably attributed to the coexistence of hydrophobic attraction and π–π-type interactions, superimposed on the same van der Waals and physical trapping effects that operate for PP and PE [53,54]. Chitosan does not contribute π-electronic character, but it enhances these interactions indirectly by dispersing the inorganic phases, maintaining structural intimacy among the components, and increasing the probability of collision and adhesion between suspended particles and the active composite surface.

The role of electrostatic effects should be considered with caution. Electrostatic attraction may contribute when the microplastic particles carry oxidized or aged surface functionalities that render their surfaces partially negative in water. Under such conditions, the protonated amino groups of chitosan and the interfacial charge environment generated by the hybrid composite may support initial particle approach and attachment. However, for pristine PP and PE, which are intrinsically nonpolar, electrostatic attraction is unlikely to be the dominant driving force. Therefore, the adsorption of these polyolefins should be described mainly in terms of hydrophobic adhesion, van der Waals forces, and physical entrapment, whereas PS benefits additionally from π-mediated interactions [55,56,57].

Overall, the outstanding adsorption behavior of ZIF-67/*g*-C_3_N_4_/CS arises from the synergistic integration of complementary functions within a single material. Chitosan furnishes a stable and hydrophilic supporting matrix, *g*-C_3_N_4_ introduces layered π-rich domains that are particularly favorable for aromatic PS capture, and ZIF-67 contributes a porous adsorption scaffold with high accessible surface area. The resulting hybrid interface is capable of efficiently engaging microplastics with different chemical structures through multiple concurrent pathways, thereby explaining both the high removal efficiencies and the performance sequence PS > PP > PE. This synergy between porous architecture, interfacial chemistry, and polymer-compatible surface functionality is central to the superior adsorption capacity of the developed nanocomposite. In addition, FTIR spectra of the ZIF-67/*g*-C_3_N_4_/CS nanocomposite recorded before and after PS adsorption are presented in Figure S2. The main characteristic absorption bands of the composite are retained after adsorption, indicating that its chemical framework remains stable during the process. However, moderate changes in band intensity and slight broadening, particularly in the O–H/N–H stretching region near 3400 cm^−1^ and within the C–N/C=N fingerprint region, suggest that surface functional groups and π-conjugated domains participate in the interaction with PS particles. These spectral variations support interfacial adsorption through hydrogen-bond-assisted interactions, hydrophobic forces, and π-associated interactions, rather than the formation of new covalent bonds.

### 3.7. Application of ZIF-67/g-C_3_N_4_/CS for the Removal of PS, PP, and PE Microplastics from Diverse Environmental Water Samples

**Figure 8:**
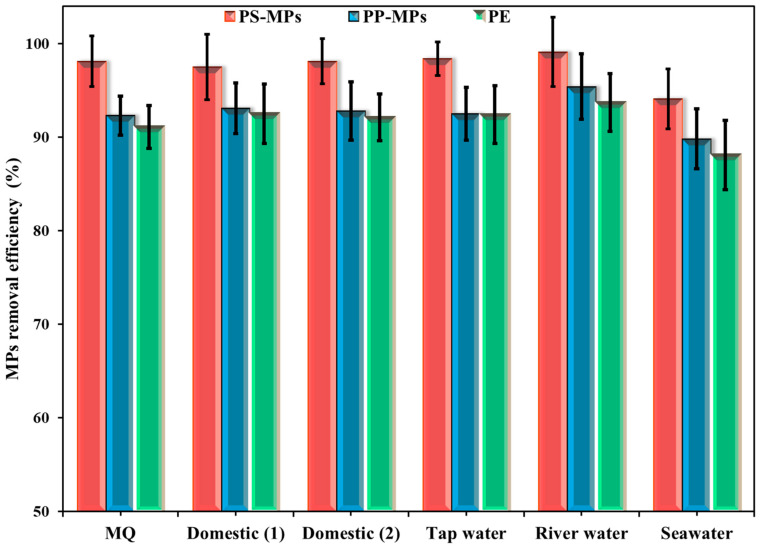
Removal efficiency of PS, PP, and PE microplastics (C_0_ = 15 mg L^−1^) by the ZIF-67/*g*-C_3_N_4_/CS nanocomposite in environmental water samples with different matrix compositions.

The performance of the ZIF-67/*g*-C_3_N_4_/CS nanocomposite was further assessed in real aqueous matrices, including seawater, river water, tap water, and two domestic wastewater samples, and the corresponding results are presented in Figure 8, while the main water-quality characteristics are summarized in Table 1. Because the pH values of the tested waters fall within a relatively narrow range (7.2–8.3), the differences in removal efficiency cannot be attributed primarily to pH variation. Instead, the observed matrix effect is more reasonably associated with differences in ionic strength, total dissolved solids, and background electrolyte composition. Despite these differences, the nanocomposite maintained high removal efficiency in all matrices, confirming that the adsorbent remains effective under chemically diverse environmental conditions.

For PS microplastics, the removal efficiency remained consistently high across all samples, with the approximate performance order river water > tap water ≈ domestic wastewater (2) > MQ water ≈ domestic wastewater (1) > seawater. A similar trend was observed for PP and PE, although at slightly lower efficiencies, and the overall adsorption sequence remained PS > PP > PE in every matrix. This behavior is consistent with the structural features of both the adsorbent and the target microplastics. The superior capture of PS arises from its aromatic phenyl-containing backbone, which can interact more favorably with the π-conjugated surface of *g*-C_3_N_4_ and the imidazolate-rich framework of ZIF-67 through π-associated dispersive interactions, in addition to hydrophobic adhesion. In contrast, PP and PE are nonpolar polyolefins lacking aromatic functionality, so their adsorption is governed predominantly by hydrophobic interaction, van der Waals attraction, and physical retention within the porous composite architecture. The role of chitosan is mainly to provide a hydrophilic, amino- and hydroxyl-rich matrix that improves particle–surface contact, disperses the inorganic phases, and stabilizes the hybrid structure in water, rather than acting as the dominant adsorption site itself.

The matrix-dependent differences shown in Figure 8 can be interpreted mainly in terms of electrolyte effects. The relatively strong performance observed in river water and tap water suggests that moderate concentrations of background ions can favor microplastic capture by reducing interparticle repulsion and improving contact between suspended particles and the composite surface. By contrast, seawater, which contains by far the highest TDS and the greatest concentrations of SO_4_^2−^, Cl^−^, Mg^2+^, Ca^2+^, K^+^, and Na^+^ (Table 1), produced the lowest removal efficiency for all three microplastics. This decrease is consistent with the strong matrix complexity of seawater, where high ionic strength can alter colloidal behavior, compress the electrical double layer, and introduce extensive competition and screening effects at the adsorbent–water interface. Under such conditions, the accessibility of favorable adsorption domains may be partially reduced, and the suspended microplastics may undergo matrix-induced changes in dispersion state that are less favorable for capture. Therefore, the lower efficiencies in seawater should be attributed primarily to its extreme salinity and multicomponent ionic background rather than to any intrinsic loss of adsorbent activity.

While naturally occurring, organic matter may contribute to the interfacial behavior of microplastics in real waters, the present dataset more clearly indicates that ionic composition and salinity are the dominant measured variables controlling matrix-dependent adsorption behavior. Even so, the nanocomposite preserved high removal performance in all samples, demonstrating substantial resistance to matrix interference.

The excellent stability of adsorption across different waters can be linked directly to the multifunctional architecture of the material. ZIF-67 contributes a porous adsorption scaffold and a chemically heterogeneous imidazolate-rich surface; *g*-C_3_N_4_ introduces layered π-rich domains that are especially beneficial for PS capture; and chitosan provides a hydrophilic polymer matrix that improves dispersion, structural integrity, and interfacial contact. The combination of these three moieties creates a robust hybrid interface capable of accommodating microplastics with different chemical structures in waters of very different composition. A one-way ANOVA yielded *p*-values exceeding 0.05 across all experimental conditions, with computed *F*-values remaining below their respective critical thresholds. This demonstrates that the variations between the investigated matrices lack statistical significance at a 95% confidence interval. This result confirms that the adsorption process remains highly reliable and reproducible even when the water chemistry changes markedly, highlighting the practical potential of ZIF-67/*g*-C_3_N_4_/CS for microplastic remediation in realistic environmental systems.

## 4. Conclusions

A cross-linked ZIF-67/*g*-C_3_N_4_/CS nanocomposite was successfully prepared for the rapid capture of PS, PP, and PE microplastics from water. The composite combined a high BET surface area of 761.5 m^2^ g^−1^ with a mesoporous structure, an average pore diameter of 11.46 nm, and a total pore volume of 0.0813 cm^3^ g^−1^. Under the optimum conditions of pH 7.6 and 25 °C, adsorption reached equilibrium within approximately 25 min, with removal efficiencies of 97.4% for PS, 92.1% for PP, and 90.3% for PE. The corresponding Langmuir maximum capacities were 97.69, 94.86, and 93.67 mg g^−1^, respectively.

The consistently higher affinity for PS was associated with its aromatic phenyl groups, which can interact with the π-conjugated domains of *g*-C_3_N_4_ and the imidazolate-rich surface of ZIF-67. In comparison, adsorption of PP and PE was governed mainly by hydrophobic adhesion, van der Waals forces, and physical retention on the rough and porous composite surface. Chitosan contributed a hydrophilic and structurally cohesive matrix that improved the dispersion of the inorganic phases and promoted contact between the suspended particles and the active surface. Control experiments with the individual components further showed that the superior performance of the ternary material resulted from their cooperative functions rather than from any component acting alone.

The nanocomposite maintained high removal efficiency in filtered and spiked environmental–water matrices despite differences in salinity and ionic composition. It also retained more than 95% PS removal over five consecutive adsorption–desorption cycles, indicating good regeneration performance under the tested batch conditions. Overall, the rapid uptake, high removal efficiency, broad polymer affinity, and favorable reusability of ZIF-67/*g*-C_3_N_4_/CS support its potential as an adsorbent for microplastic removal and wastewater-polishing applications. Further evaluation under continuous-flow conditions, together with attrition, leaching, and long-term stability tests, will be required to establish its suitability for larger-scale treatment systems.

## Figures and Tables

**Table 1:** Water quality characteristics of the environmental samples used to evaluate PS/PP/PE microplastic removal by the ZIF-67/*g*-C_3_N_4_/CS nanocomposite.

Parameter	Tap Water	Domestic Wastewater (1)	Domestic Wastewater (2)	River Water	Seawater
TDS (mg L^−1^)	284 ± 75	917 ± 96	965 ± 105	126 ± 43	34,861 ± 153
pH	7.15 ± 0.08	7.22 ± 0.05	7.85 ± 0.03	7.22 ± 0.05	8.31 ± 0.02
Cl^−^ (mg L^−1^)	11.52 ± 2.5	2.19 ± 0.02	1.53 ± 0.05	6.33 ± 1.41	19,352 ± 410
NO_3_^−^ (mg L^−1^)	4.93 ± 1.22	1.52 ± 0.07	5.3 ± 1.1	11.50 ± 1.53	ND *
SO_4_^2−^ (mg L^−1^)	6.81 ± 1.52	16.43 ± 3.32	15.91 ± 3.21	8.13 ± 1.55	2518.5 ± 357
Na^+^ (mg L^−1^)	6.14 ± 0.75	39.51 ± 4.35	44.62 ± 2.17	5.35 ± 0.81	10,016 ± 257
K^+^ (mg L^−1^)	1.8 ± 0.03	18.22 ± 5.1	13.41 ± 2.2	3.14 ± 1.4	394 ± 32
Ca^2+^ (mg L^−1^)	7.18 ± 2.7	35.11 ± 9.5	42.71 ± 6.3	11.45 ± 2.5	513 ± 73
Mg^2+^ (mg L^−1^)	1.83 ± 0.08	64.7 ± 1.5	71.5 ± 1.7	4.15 ± 0.87	1274 ± 315

* ND: Not detected.

**Table 2:** Non-linear kinetic equations for the sorption of PS, PP and PE on ZIF-67/*g*-C_3_N_4_/CS nanocomposite at 25.0 °C, for 15 mg L^−1^ as initial concentration, pH 7.6 and 50 mg nanocomposite mass [50,51].

Kinetic Model	Sorption Non-Linear Kinetic Equations	Calculated Parameters	PS	PP	PE
PFO	$q_{t}=q_{e}\;\cdot \;(1-e^{-k_{1}t})$	*q*_e_ (mg g^−1^)	24.22	22.41	21.28
		*k*_1_ (min^−1^)	0.1511	0.1862	0.1706
		*R* ^2^	0.7901	0.6803	0.7839
		*RMSE* (mg g^−1^)	1.557	1.334	1.559
PSO	$q_{t}=\frac{k_{2}q_{e}^{2}\;t}{{1+k}_{2}q_{e}\;t}$	*q*_e_ (mg g^−1^)	26.78	24.25	23.33
		*k*_2_ (g mg^−1^ min^−1^)	9.3 × 10^−3^	1.4 × 10^−2^	1.2 × 10^−2^
		*R* ^2^	0.915	0.9276	0.9322
		*RMSE* (mg g^−1^)	1.071	1.012	1.052
Elovich	$q_{t}=\frac{1}{\beta }\;\ln(\alpha \;\beta )+\frac{1}{\beta }\ln(t)$	*α* (mg g^−1^ min^−1^)	26.62	46.40	30.42
		*β* (g mg^−1^)	0.2214	0.2694	0.2642
		*R* ^2^	0.9013	0.9032	0.9214
		*RMSE* (mg g^−1^)	1.391	1.333	1.226

*q*_e_ and *q*_t_ (mg g^−1^) are the adsorption capacity at equilibrium and at time *t*, respectively. *k*_1_ and *k*_2_ are the PFO and PSO adsorption rate constants of kinetic models. α relates to the initial adsorption rate and β is the desorption rate constant.

**Table 3:** Non-linear Langmuir and Freundlich isotherm parameters modeled for the equilibrium adsorption of PS, PP, and PE microplastics, onto ZIF-67/*g*-C_3_N_4_/CS nanocomposite (initial concentration of MPs is 5–50 mg L^−1^, 50 mg nanocomposite mass, pH 7.6 at 25.0 °C) [52,53].

Isotherm Model and Parameters	PS	PP	PE
Langmuir model: $q_{e}=\frac{q_{m}K_{L}C_{e}}{1+K_{L}C_{e}}$			
*q*_m_ (mg g^−1^)	97.69	94.86	93.67
*K*_L_ (L mg^−1^)	0.02424	0.02767	0.02389
*R* ^2^	0.9501	0.9535	0.9580
*R_L_*	0.4521	0.4196	0.4557
*RMSE*	1.283	1.202	1.144
Freundlich model: qe=KF Ce1n			
*K*_F_ (mg^1−n^·L^n^·g^−1^)	4.451	5.123	4.205
*n*	1.552	1.623	1.547
*R* ^2^	0.9160	0.9152	0.9270
*RMSE*	1.558	1.622	1.948

## Data Availability

The original contributions presented in this study are included in the article/Supplementary Material. Further inquiries can be directed to the corresponding author.

## Supplementary Materials

The following supporting information can be downloaded at: https://www.mdpi.com/article/10.3390/polym18151904/s1, Figure S1. Nitrogen adsorption–desorption isotherm of the ZIF-67/*g*-C_3_N_4_/CS nanocomposite measured at 77.40 K; Figure S2. FTIR of the ZIF-67/*g*-C_3_N_4_/CS nanocomposite before and after the PS-MPs adsorption.
