# Supplementary material for: High-Efficiency Adsorption of PS, PE, and PP Microplastics from Environmental Waters Using a Cross-Linked Chitosan/Graphitic Carbon Nitride/ZIF-67 Nanocomposite
Source: Polymers (Basel). 2026 Aug 3;18(15):1904. doi: 10.3390/polym18151904 (PMC13468130; doi:10.3390/polym18151904)
Supplement: Supplementary file 1 [file polymers-18-01904-s001.zip › polymers-4408939-supplementary.pdf]

Supplementary materials:

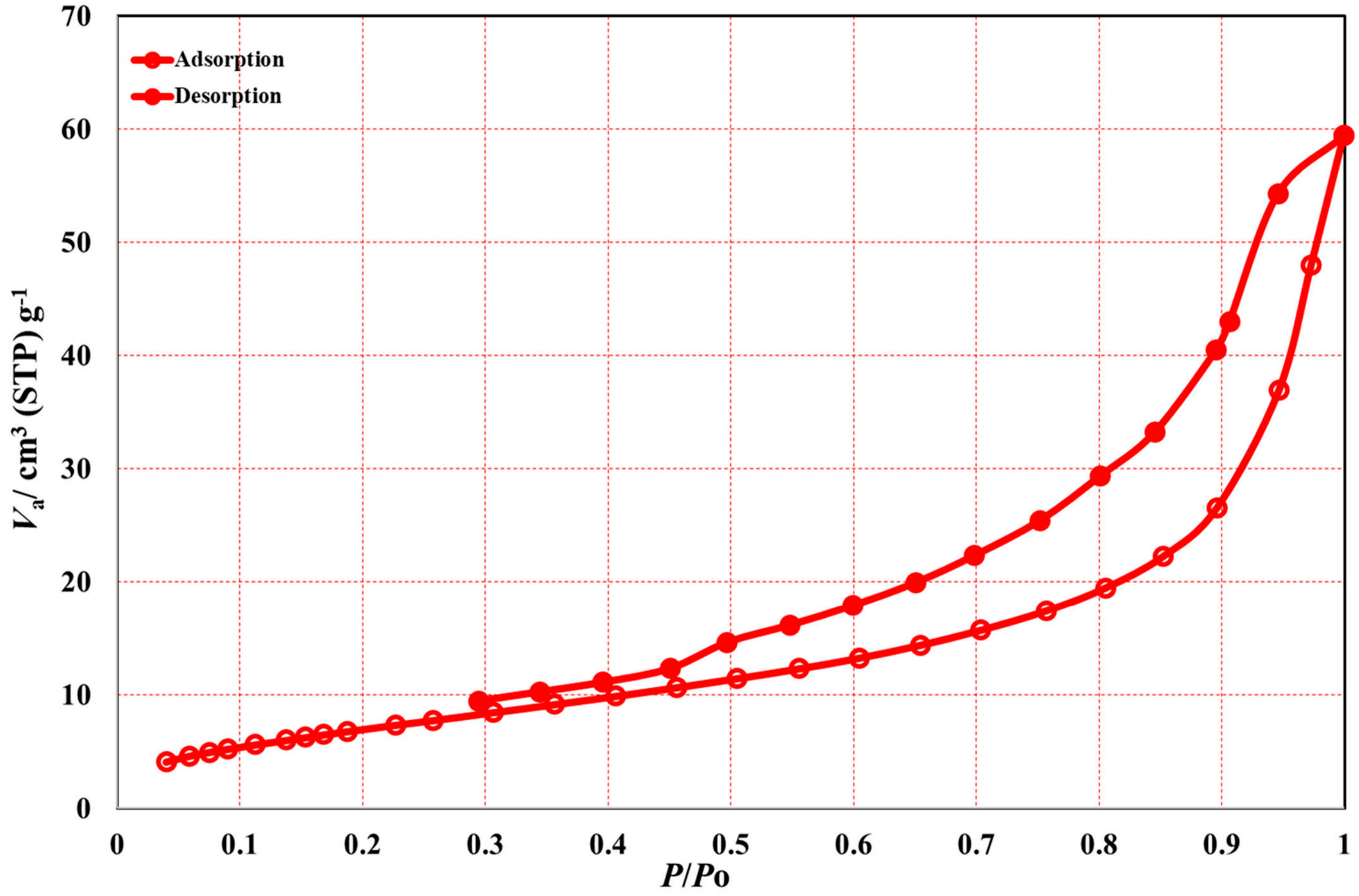

Figure S1. Nitrogen adsorption–desorption isotherm of the ZIF-67/*g*-C_3N_4/CS nanocomposite measured at 77.40 K.

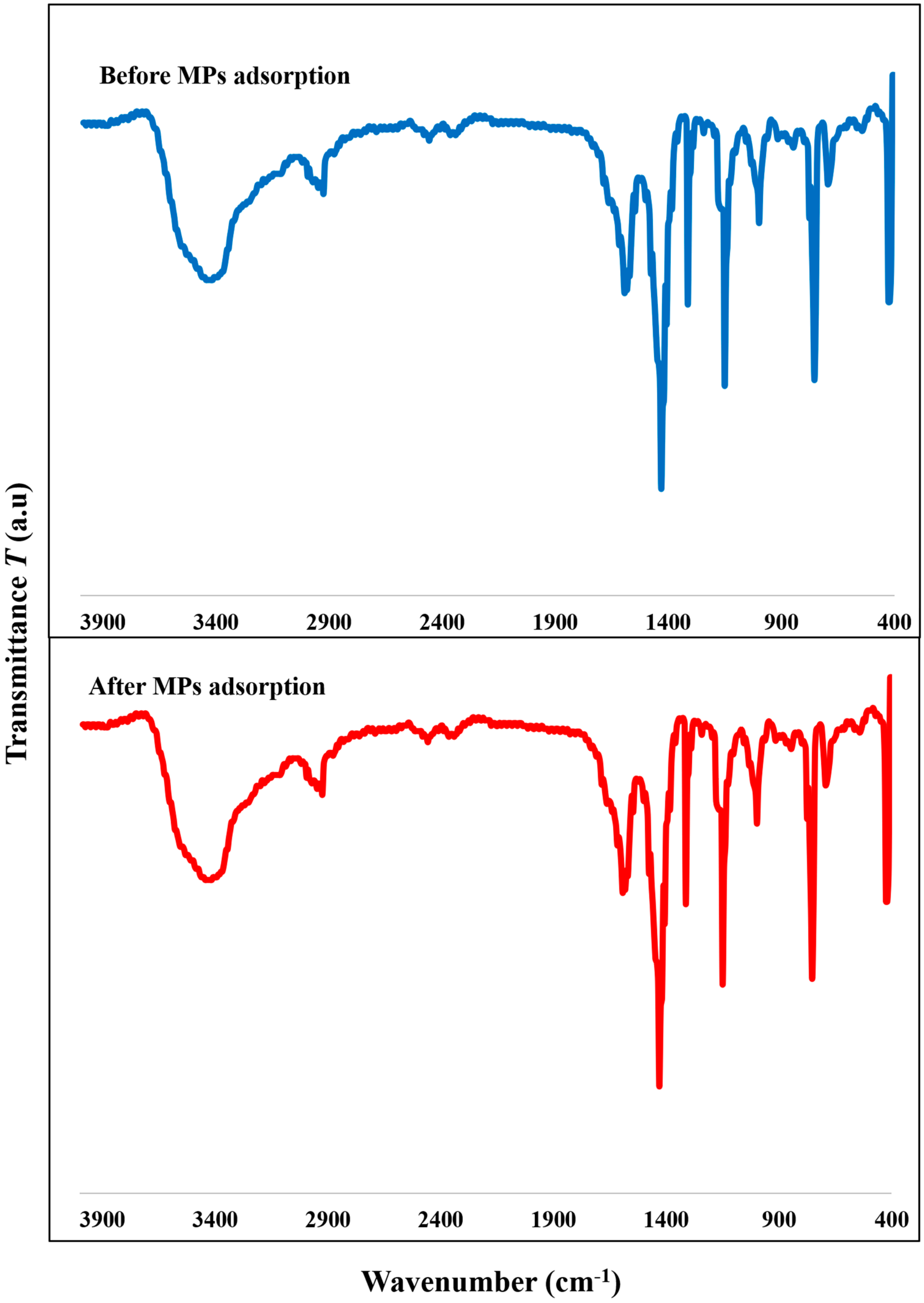

Figure S2. FTIR of the ZIF-67/*g*-C_3N_4/CS nanocomposite before and after the PS-MPs adsorption.
